# Attenuated pupillary light responses and downregulation of opsin expression parallel decline in circadian disruption in two different mouse models of Huntington’s disease

**DOI:** 10.1093/hmg/ddw359

**Published:** 2016-11-27

**Authors:** Koliane Ouk, Steven Hughes, Carina A. Pothecary, Stuart N. Peirson, A. Jennifer Morton

**Affiliations:** 1 Department of Physiology, Development and Neuroscience, University of Cambridge, Cambridge, UK; 2 Nuffield Department of Clinical Neurosciences, Sleep and Circadian Neuroscience Institute, University of Oxford, Oxford, UK

## Abstract

Circadian deficits in Huntington’s disease (HD) are recapitulated in both fragment (R6/2) and full-length (Q175) mouse models of HD. Circadian rhythms are regulated by the suprachiasmatic nuclei (SCN) in the hypothalamus, which are primarily entrained by light detected by the retina. The SCN receives input from intrinsically photosensitive retinal ganglion cells (ipRGCs) that express the photopigment melanopsin, but also receive input from rods and cones. In turn, ipRGCs mediate a range of non-image forming responses to light including circadian entrainment and the pupillary light response (PLR). Retinal degeneration/dysfunction has been described previously in R6/2 mice. We investigated, therefore, whether or not circadian disruption in HD mice is due to abnormalities in retinal photoreception. We measured the expression of melanopsin, rhodopsin and cone opsin, as well as other retinal markers (tyrosine hydroxylase, calbindin, PKCα and Brna3), in R6/2 and Q175 mice at different stages of disease. We also measured the PLR as a ‘readout’ for ipRGC function and a marker of light reception by the retina. We found that the PLR was attenuated in both lines of HD mice. This was accompanied by a progressive downregulation of cone opsin and melanopsin expression. We suggest that disease-related changes in photoreception by the retina contribute to the progressive dysregulation of circadian rhythmicity and entrainment seen in HD mice. Colour vision is abnormal in HD patients. Therefore, if retinal deficits similar to those seen in HD mice are confirmed in patients, specifically designed light therapy may be an effective strategy to improve circadian dysfunction.

## Introduction

Huntington’s disease (HD) is an inherited neurodegenerative disorder caused by an abnormal CAG expansion in the HD gene (1). HD is characterised by progressive motor, cognitive and psychiatric disturbances (2). Sleep disturbances and circadian abnormalities are also common features of HD (3–7), and are recapitulated in a number of different mouse models (8–12).

In mammals, the suprachiasmatic nuclei (SCN) of the hypothalamus are the site of the master pacemaker regulating circadian rhythms in physiology and behaviour (13,14). The light detected by the retina is the primary cue for the entrainment of the SCN oscillator to the environmental light/dark cycle (15). Innervation of the SCN via the retinohypothalamic tract (16) is primarily from melanopsin-expressing intrinsically photosensitive retinal ganglion cells (ipRGCs). Furthermore, ipRGCs are also involved in the regulation of other non-image forming responses to light, including the pupillary light response (PLR), sleep induction and pineal melatonin release (17–19). Whilst rods and cones are critically important for vision, they were initially thought not to be necessary for circadian photoentrainment (20,21). However, mice lacking melanopsin can still entrain to light/dark cycles, although they show deficits in phase shifting and pupillary responses (19,22,23). By contrast, mice lacking rods, cones and melanopsin no longer respond to light (24). As such, rods and cones may also provide contributions to non-image forming responses under certain conditions (22,25–27). These observations are explained by the fact that ipRGCs also receive inputs from rods and cones. Selective ablation of ipRGCs results in a loss of photoentrainment, showing that these cells act as a conduit for light input to the SCN (28).

Melanopsin ipRGCs have not been investigated in the retina of either HD patients or mice. Given the prominent role of melanopsin ipRGCs in mediating circadian photoentrainment, we investigated whether progressive neurodegeneration of ipRGCs, loss of the rod or cone inputs, or loss of function of melanopsin contribute to the progressive dysregulation of circadian rhythmicity and impairment of photic synchronization in HD. Such loss may contribute to abnormal circadian entrainment phenotype (such as that seen in HD mice), whereas loss of all photoreception from the retina would result in free-running rather than arrhythmia or rhythm fragmentation. For our studies, we used a well characterised transgenic fragment HD mouse model (R6/2 line) that shows a severe and rapid progression of disease (3,29,30). We have shown previously that bright light therapy has beneficial effects on behavioural deficits in R6/2 mice (31). Bright light therapy may be effective in treating the circadian disruption in R6/2 mice because the light input pathways become impaired. We also used a ‘knock-in’ HD mouse (Q175), that carries the HD mutation in a genetically-relevant context, and recapitulates the HD circadian and sleep phenotype in a slower fashion (9,10).

Neurodegeneration caused by mutant huntingtin first affects neurons in the striatum and cerebral cortex (32), and then extends to all brain areas as the disease progresses (33). Interestingly, mutant huntingtin protein aggregates (inclusions) have been described in the retina of R6/2 mice as well as a related R6/1 line (34,35). These inclusions were accompanied by retinal degeneration and functional impairment in both lines of mice at the end stage (34–37). We hypothesised that retinal deficits that cause visual defects might also contribute to the dysregulation of circadian rhythmicity. Unfortunately, the literature regarding HD human retinal pathology is not only sparse but also conflicting. A *post mortem* examination of the eyes of a single HD patient with advanced disease did not reveal any histological abnormalities (38). By contrast, cone function has been reported to be impaired in patients performing visual tasks (39). Furthermore a recent study revealed abnormalities in colour vision in HD patients who had reduced thickness of the temporal part of the retinal nerve fibre layer (RNFL), suggesting the involvement of the retina in the neurodegenerative process of HD (40).

Our working hypothesis is that the deterioration of melanopsin pathways in HD may play a role in the circadian disruption seen in HD patients. To address this, we measured the PLR (that represents a readout for ipRGC functionality and a marker of retinal sensitivity), and performed a detailed immunohistochemical study of the HD retina. We assessed the expression of key photopigments (melanopsin, ultraviolet sensitive (UVS) and middle-wave sensitive (MWS) cone opsins and rhodopsin), markers of other retinal cell types (tyrosine hydroxylase (TH), calbindin, Brain-specific homeobox/POU domain protein 3a (Brn3a) and protein kinase C alpha (PKCα)) and we examined the occurrence of huntingtin (Htt) depositions (using MW8), in retina from R6/2 and Q175 mice at different stages of the disease.

## Results

### Pupil light responses are progressively affected in R6/2 mice

**Figure 1.**
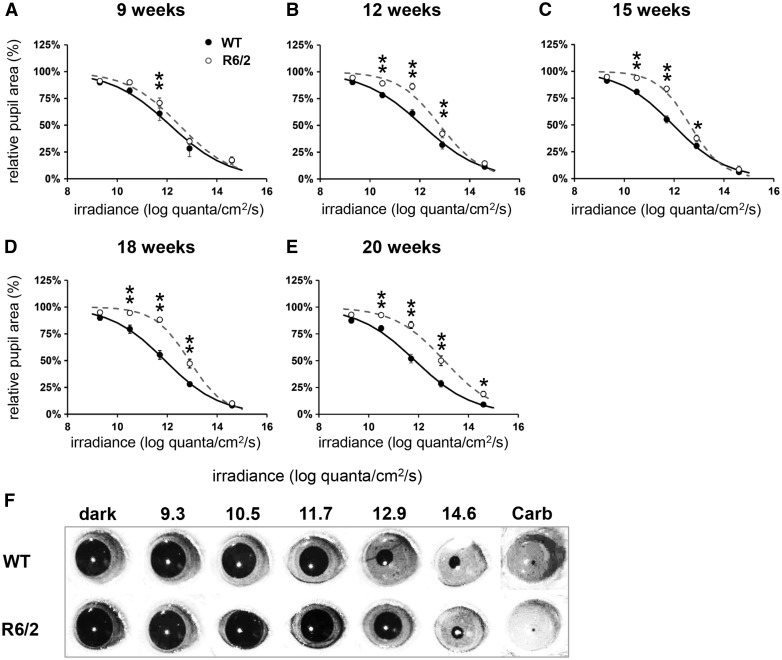
Progressive deficits in pupillary responses to light in R6/2 mice. A single light exposure (10 s, 470 nm) was tested at five different irradiances (9.3, 10.5, 11.7, 12.9 and 14.6 log quanta/cm^2^/s) to test consensual pupillary responses. (**A–E**) Phase response curves to light irradiance are shown from wild type (WT, black symbols and solid line) and R6/2 (white symbols and dashed line) mice at 9, 12, 15, 18 and 20 weeks. (F) Representative images of pupil size from R6/2 and WT mice at 20 weeks, recorded before the light stimulus in total darkness (dark) and at maximal constriction in response to a 10-s light exposure at 9.3, 10.5, 11.7, 12.9 and 14.6 log quanta/cm^2^/s, and 10 min after application of carbachol (Carb). Data shown in A-E are the mean maximal pupil constriction observed following light exposure from 19 to 20 mice ± SEM. Curves are fitted to a sigmoid function. Where error bars are not visible, they are obscured by the symbols. *P < 0.05, **P < 0.01.

In order to assess retina function and ipRGC output in wild type (WT) and R6/2 mice, we stimulated one eye with a 10-s light stimulus and recorded the PLR in the other eye, at 9, 12, 15, 18 and 20 weeks of age (Fig. 1). We tested five irradiances, ranging from a bright light stimulus at 14.6 log quanta/cm^2^/s that induced near complete consensual pupil constriction to a dim light at 9.3 log quanta/cm^2^/s that induced almost no consensual pupil response in normal WT mice. Irradiance response curves were then constructed for WT and R6/2 mice at each age.

**Figure 2.**
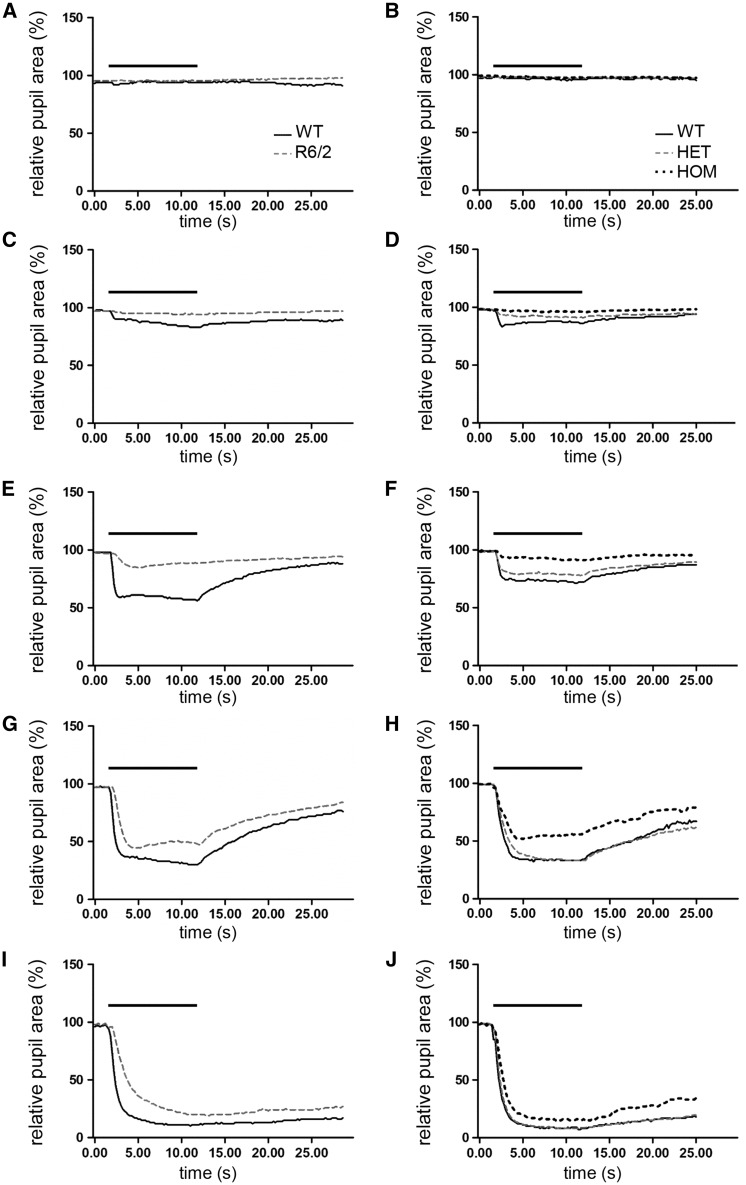
Dynamics of pupil light reflex in R6/2 and homozygous Q175 mice at late stage of lifespan. Consensual pupil constriction profiles shown are recorded from 20 week old R6/2 (**A,C,E,G,I**) and 20 month old Q175 homozygous (HOM) (**B,D,F,H,J**) mice in response to 10-s light exposure at different illumination irradiance: 9.3 (A, B), 10.5 (C, D), 11.7 (E, F), 12.9 (G, H) and 14.6 (I, **J**) log quanta/cm^2^/s. The bar above the traces indicate the duration of light stimuli (from 2 to 12 s). All data are mean of response ± SEM.

We observed an age-dependent decline in the PLR of R6/2 mice, but not in age-matched WT controls. We found a significant effect of irradiance on the magnitude of pupil constriction in WT and R6/2 mice at all ages [*F*_(4,732) _= 2103.28; *P <* 0.001; Fig. 1A–E]. There were also significant age- and genotype-related changes in PLR in interaction with irradiance across the longitudinal study [light irradiance x age, *F*_(4,732) _= 36.21; *P <* 0.001 and irradiance x genotype, *F*_(16,732) _= 3.24; *P <* 0.001]. At 9 weeks, there was already a significant effect of genotype on PLR [*F*_(1,37) _= 6.73, *P <* 0.05; Fig. 1A], with presymptomatic R6/2 mice showing an attenuation of their response to light under low irradiance at 11.6 log quanta/cm^2^/s compared to WT mice (*P <* 0.01). At 12 weeks, there was an effect of genotype on PLR [main effect: *F*_(1,38) _= 28.72, *P <* 0.001] as well as interaction between this factor and irradiances [*F*_(4,152) _= 6.46, *P <* 0.001; Fig. 1B]. *Post hoc* analysis revealed that the attenuation in PLR seen earlier at 11.6 log quanta/cm^2^/s compared to WT mice was maintained at 12 weeks (*P <* 0.01). At this age, PLR deficits were also seen at 10.5 and 12.9 log quanta/cm^2^/s (*P <* 0.01). At 15 weeks, the PLR deficits were maintained compared to age-matched WT mice at 10.5, 11.6 and 12.9 log quanta/cm^2^/s (Fig. 1C). Finally, while the R6/2 retina at late symptomatic stages (18 and 20 weeks) was still able to constrict to 11.6 and 12.9 but not to 10.5 log quanta/cm^2^/s, the corresponding PLR response was severely attenuated compared to WT mice (*P <* 0.01; Figs. 1D and E; 2C, E, and G). Interestingly, the ability to achieve maximal pupil constriction was only affected in the oldest R6/2 mice tested (Fig. 1E), where R6/2 mice at 20 weeks showed a significant attenuation in PLR under bright light condition at 14.6 log quanta/cm^2^/s (*P <* 0.05). As presented in Fig. 2I, the kinetic profile of the PLR showed a slower initial phase of constriction in R6/2 mice (maximal constriction at 11s) compared to WT mice (6s). It is notable that almost half of 20 week old R6/2 mice (8 out of 20) developed ptosis, characterised by the drooping of the upper eyelid.

To verify whether or not there was an intrinsic defect in the iris sphincter pupillae muscle of the R6/2 mice, a carbachol test was performed (Fig. 1F). Both WT and R6/2 retinas were fully responsive to carbachol that induced full pupil constriction in all mice. Thus, the muscles responsible for driving pupil constriction were not affected, confirming that the changes in PLR seen in R6/2 mice were not due to the inability of the pupil to constrict.

### Pupil light responses are attenuated in 20 month old Q175 mice

**Figure 3.**
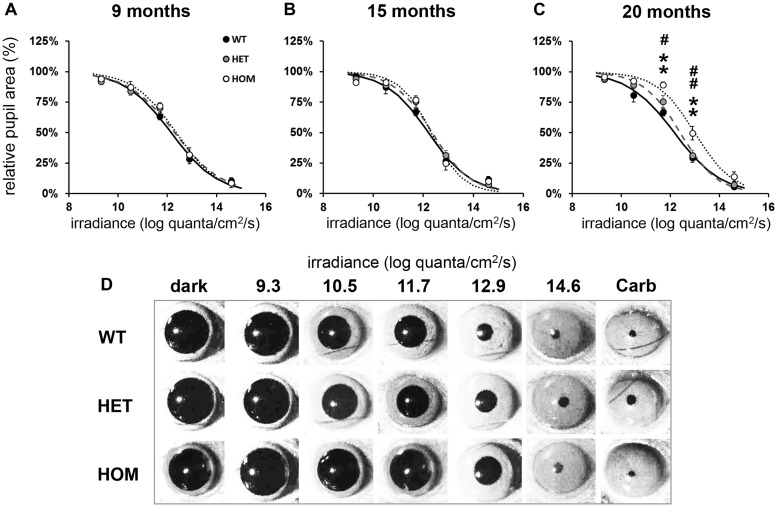
Defects in pupillary responses to light in 20 month old homozygous Q175 mice. A single light exposure (10 s, 470 nm) was tested at five different irradiances (9.3, 10.5, 11.7, 12.9 and 14.6 log quanta/cm^2^/s) to induce consensual pupillary constriction. **(A-C)** Phase response curves to light irradiance are shown from wild type (WT, black symbols and solid line), Q175 heterozygous (HET, grey symbols and dashed line) and Q175 homozygous (HOM, white symbols and dotted line) mice at 9, 15 and 20 months. **(D)** Representative images of pupil area from Q175 and WT mice at 20 months, recorded in dark and at maximal constriction in response to a 10-s light exposure at 9.3, 10.5, 11.7, 12.9 and 14.6 log quanta/cm^2^/s, and 10 min after application of carbachol (Carb). All data in A-C are mean maximal pupil constriction observed following light exposure from 6 to 12 mice ± SEM. Curves are fitted to a sigmoid function. Where error bars are not visible, they are obscured by the symbols. Asterisks show significant difference between Q175 HOM and WT mice, **P < 0.01. Hashes show significant differences between Q175 HOM and HET mice, #*P <* 0.05 and ##*P <* 0.01. There were no statistical differences between response of Q175 HET and WT mice.

To determine whether or not the PLR deficits seen in R6/2 mice were recapitulated in a mouse model of HD with a slower progression of disease, we tested Q175 mice at 9, 15 and 20 months of age using the same protocol (Fig. 3).

We observed a decline in PLR only in 20 month old Q175 homozygous (HOM) mice (Figs. 2D, F, H and J; 3C), while there was no change in heterozygous (HET) and WT mice at any age tested. We found, however, regardless of the age tested, an overall effect of light irradiance [*F*_(4,260) _= 1174.86; *P <* 0.01] and genotype [*F*_(2,65) _= 4.49; *P <* 0.05] on the magnitude of PLR in WT and Q175 mice, as well as a significant interaction between these factors [*F*_(8,260) _= 1.98; *P <* 0.05; Fig. 3]. Further analysis of the data at 9 months or 15 months did not show any main effect of genotype in the PLR between Q175 HET, Q175 HOM or WT mice [*F*_(2,17) _= 0.19; *P >* 0.05; (Fig. 3A) and *F*_(2,24) _= 0.36; *P >* 0.05; (Fig. 3B), respectively]. At 20 months however, we found a significant effect of genotype [*F*_(2,24) _= 9.36; *P <* 0.001], light irradiance [*F*_(4,96) _= 423.20; *P <* 0.001] on the PLR, and also in the interaction between these factors [*F*_(8,96) _= 2.35; *P <* 0.05; Fig. 3C]. *Post hoc* analysis revealed significant PLR deficits in HOM mice at 11.6 and 12.9 log quanta/cm^2^/s compared to both WT and HET mice. However, no differences were observed in response to bright light stimuli, with levels of pupil constriction similar between HOM and WT mice at 14.6 log quanta/cm^2^/s. No differences were found between HET and WT mice. The kinetic profile of the PLR presented in Fig. 2J shows an initial phase of constriction similar between HOM and age-matched WT mice (with maximal pupil constriction reached at 8.4 and 7.4s, respectively), but HOM mice had a slightly more rapid recovery of pupil size compared to WT mice. It is notable that half of the 20 month old Q175 HOM mice (4 out of 8) developed ptosis.

As with WT mice, Q175 HET and Q175 HOM pupils fully constricted in response to carbachol confirming that the muscles responsible of the pupil constriction were not affected (Fig. 3D).

Overall, the PLR phenotype was similar between R6/2 and Q175 mice. For both models, attenuated PLR was observed at low and moderate intensities of light, with full pupil constriction observed to bright light stimuli at all-time points, with the exception of the oldest R6/2 mice tested. This phenotype is inconsistent with a deficit in melanopsin alone, as the PLR phenotype of melanopsin-deficient (*Opn4^-/-^*) mice is characterised by a failure to reach full constriction to bright light stimuli with normal constriction observed at moderate and low intensities of light. The PLR phenotype observed in R6/2 and Q175 mice is therefore likely to be caused, at least in part, by a functional change in rod- and cone-driven signals, or potentially other structural changes in the PLR machinery. Changes in the onset kinetics of PLR responses would also indicate a change in rod- and cone-driven signals, as the sluggish responses of melanopsin make it unlikely that this photopigment contributes to the initial rapid phase of pupil constriction.

### Immunostaining of rods, cones and melanopsin in R6/2 retina

**Figure 4.**
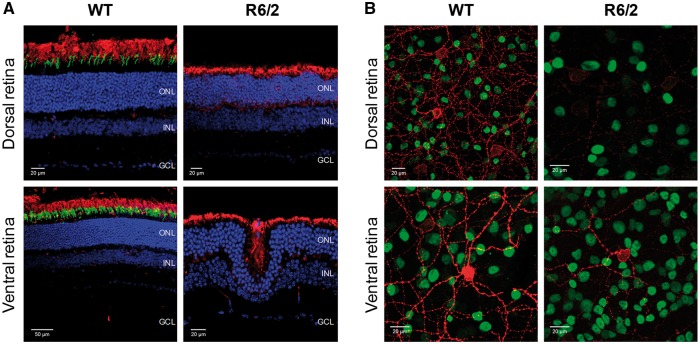
Downregulation of cone opsin and melanopsin expression in R6/2 retina at late stage of disease. Images collected from the dorsal and ventral regions of retina from 20 week old WT (A, left panels; B, left panels) and R6/2 (A, right panels; B, right panels) mice following labelling with anti-rhodopsin (red) and anti-UVS cone opsin (green) to label rods and cones (**A**) and anti-melanopsin (red) and anti-Brn3a (green) to label ipRGCs and RGCs (**B**). DAPI nuclear counterstain is shown in blue. ONL, outer nuclear layer; INL, inner nuclear layer; GCL, ganglion cell layer.

To determine the basis of the PLR phenotype observed in R6/2 and Q175 mice, we used immunostaining to perform a detailed analysis of the retina of these mice, focusing primarily on the number and distribution of rods, cones and melanopsin-expressing ganglion cells. Initial examination of 20 week old R6/2 retina (*n =* 6 R6/2 and *n =* 6 WT) showed a number of clear abnormalities compared to WT littermate controls (Fig. 4A and B). 20 week old R6/2 mice showed an extensive degree of retinal dysplasia, with the outer layers of the retina clearly invaginating into the inner layers (Fig. 4A). These anatomical changes were accompanied by a complete loss of cone opsin staining across the retina (Fig. 4A), and a widespread downregulation of melanopsin expression which was observed for all subtypes of ipRGCs (Fig. 4B). Loss of cone opsin expression was also accompanied by signs of cone degeneration in the few remaining cones identified, such as loss of outer segments and mislocalisation of opsin to the cell body. There was no indication of a widespread loss of rod photoreceptors in 20 week old R6/2 retina, although there are signs of rhodopsin protein mislocalisation (an early sign of rod degeneration), with rhodopsin staining now also evident around the cell bodies located within the outer nuclear layer (Fig. 4A).

Consistent with previous reports (41), a dorsal ventral gradient in melanopsin expression was observed in the retina from WT mice (Fig. 4B; Supplementary Material, Fig. S1A). A similar patterning of melanopsin expression was also evident in 20 week old R6/2 mice retina, although there was a clear and consistent reduction in levels of melanopsin staining observed across the retina compared to that seen in WT controls (Fig. 4B; Supplementary Material, Fig. S1B). Levels of Brn3a staining, and the total numbers of retinal ganglion cells (RGCs) were not obviously different between 20 week old R6/2 retina and WT controls (Fig. 4A and B).

**Figure 5.**
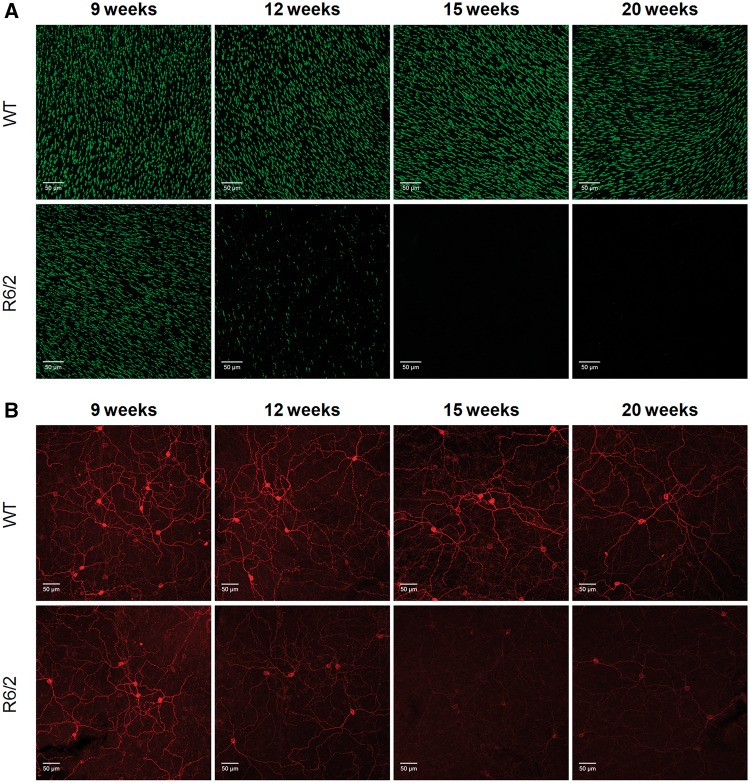
Time course of cone opsin and melanopsin expression in R6/2 mice. (**A**) Images showing the levels of UVS cone opsin observed in flatmount retina from WT and R6/2 mice at 9, 12, 15 and 20 week of age. All images collected from the ventral retina where all cones express UVS opsin. (**B**) Images showing the levels of melanopsin observed in flatmount retina from WT and R6/2 mice at 9, 12, 15 and 20 weeks of age. All images collected from the ventral retina. The levels of UVS cone opsin and melanopsin observed in the dorsal and ventral retina, as well as the expression of UVS and MWS cone opsin, is shown in Supplementary Materials, Figs S1, 2 and 4). Quantification of UVS opsin and melanopsin immunofluorescence and qPCR analysis of UVS opsin and melanopsin mRNA expression in R6/2 mice are shown in Supplementary Material, Fig. S9.

Following the initial observations from retina of 20 week old R6/2 mice, we next used immunocytochemistry and quantitative polymerase chain reaction (qPCR) analysis to examine the progression of the retina phenotype in R6/2 and WT mice aged 9 weeks, 12 weeks, 15 weeks and again 20 weeks (*n =* 4 for ICC, *n =* 5 for qPCR) (Fig. 5; Supplementary Materials, Figs. S1–S5). Additionally, we examined retina from Q175 mice (HOM, HET and WT littermates) at 20 months (*n =* 3 for ICC, *n =* 5 for qPCR) (Fig. 7; Supplementary Materials, Figs. S7–S9). Downregulation of cone opsin and melanopsin expression was evident in both R6/2 and Q175 mice.

Levels of UVS cone opsin expression were normal in R6/2 mice at 9 weeks of age, but rapidly declined by 12 weeks, with a complete loss of UVS opsin staining evident by 15 weeks (Fig. 5A; Supplementary Materials, Figs. S2B and S5D). A similar timeline was observed for the downregulation of both UVS and MWS cone opsins (Supplementary Material, Fig. S3), and also UVS opsin mRNA (Supplementary Material, Fig. S5A). By comparison, WT mice showed no significant changes in levels of cone opsin staining at any time point investigated (Fig. 5A), and cone opsin staining was entirely consistent with previous reports of dorsal ventral gradients of both UVS and MWS cone opsin expression in the mouse retina (41,42; Supplementary Material, Figs. S2 and S3).

Examination of UVS opsin staining in R6/2 mice at 12 weeks indicated not only a downregulation of cone opsin expression but also signs of cone degeneration, indicated by both morphological changes and mislocalization of cone opsin to cell bodies. To confirm that changes in opsin expression reflect cone degeneration, we also studied the expression of cone arrestin and performed labelling with peanut agglutinin (PNA) lectin that specifically labels cone cell membranes (Supplementary Material, Fig. S4). Expression of cone arrestin is downregulated by 9 weeks of age in the R6/2 retina compared to WT controls and is completely absent by 12 weeks, preceding the loss of cone opsin expression (Supplementary Material, Fig. S4A). By contrast PNA labelling of cone cell membranes was evident in R6/2 mice at 15 weeks in the absence of UVS opsin staining, indicating that loss of cone opsin and cone arrestin expression occurs prior to cone degeneration (Supplementary Material, Fig. S4B). Widespread disruption of PNA labelling and degeneration of cone cell bodies was not observed in R6/2 mice until 20 weeks of age (Supplementary Material, Fig. S4B).

In keeping with our initial findings, we observed a time-dependent decrease in melanopsin expression in R6/2 mice (Fig. 5B; Supplementary Materials, Figs. S1 and S5). Melanopsin expression levels were downregulated by 12 weeks of age, and reduced further with age. At 15 week and 20 week time points, melanopsin staining was markedly reduced, and close to levels of background staining (Fig. 5B; Supplementary Material, Fig. S1). In contrast to the clear evidence of degeneration observed for cone photoreceptors, however, there were no obvious signs of ipRGC degeneration in R6/2 mice. By increasing laser power and levels of signal gain during confocal image acquisition, it was possible to observe melanopsin expressing cells within aged R6/2 retina. In all cases where melanopsin could be visualised, the anatomy and morphology of ipRGCs appeared normal in R6/2 retina up to 15–20 weeks, albeit with markedly lower levels of melanopsin expression than was seen in WT controls. Levels of melanopsin expression remained similar in WT mice at all ages tested (Fig. 5B; Supplementary Material, Fig. S1), with the number and distribution of ipRGCs consistent with previous studies using similar approaches (41). Consistent with these observations, qPCR analysis showed a downregulation of melanopsin mRNA in R6/2 mice over time, although this effect was less dramatic than the reduction in melanopsin immunostaining (Supplementary Material, Fig. S5B and E).

To determine if Htt aggregates occur in ipRGCs, double immunostaining of aggregated Htt and melanopsin was performed. Levels of aggregated Htt detected in R6/2 retina show a steady increase over time, with high levels of aggregated Htt detected in all retinal layers at 20 weeks (Supplementary Material, Fig. S6A). Interestingly, Htt labelling was found to be almost completely absent from ipRGCs in R6/2 mice at 15 weeks, despite the presence of Htt aggregates within the vast majority of cells in the ganglion cell layer (and other retinal layers) at this time point. Aggregated Htt was, however, routinely detected within ipRGCs of R6/2 mice at 20 weeks (Supplementary Material, Fig. S6B).

**Figure 6.**
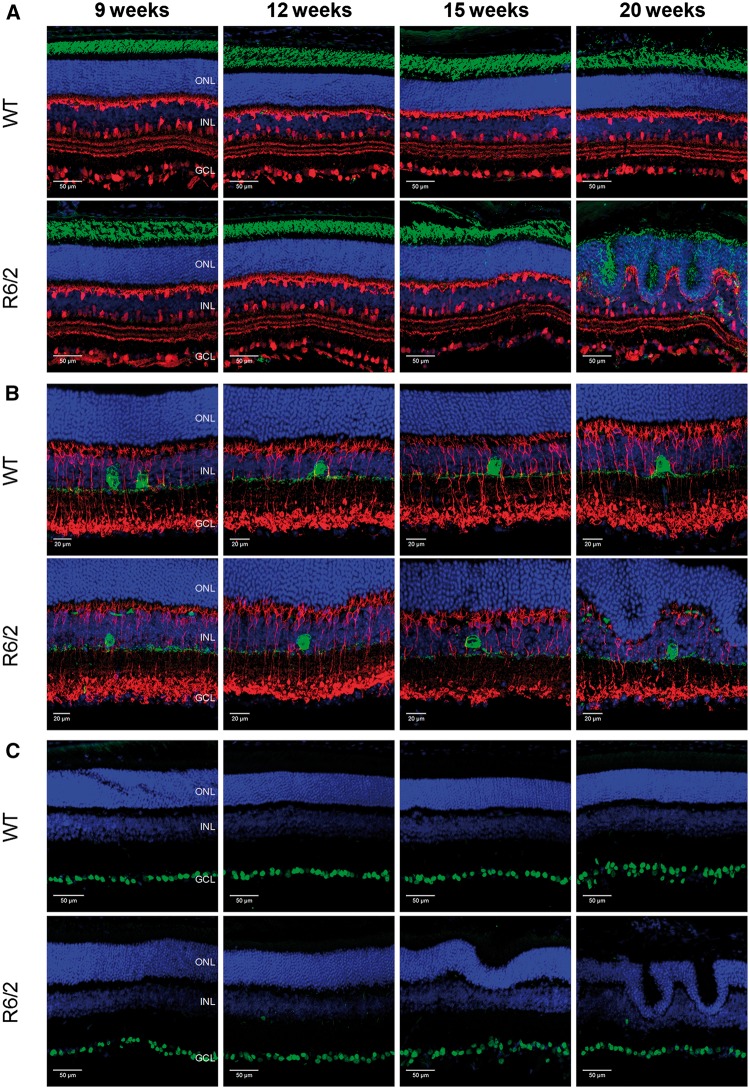
Rods, ON bipolar cells, horizontal cells, amacrine cells and retinal ganglion cells are unaffected in R6/2 mice. (**A**) Images showing labelling of anti-rhodopsin (green, labels rods) and anti-calbindin (red, labels horizontal cells and a subset of amacrine cells) in WT and R6/2 retina at 9, 12, 15 and 20 weeks of age. (**B**) Images showing labelling of anti-protein kinase-C alpha (PKCα) (red, labels ON bipolar cells) and anti-tyrosine hydroxylase (TH) (green, labels dopaminergic amacrine cells) in WT and R6/2 retina. (**C**) Images showing labelling of anti-Brn3a (green, marker of retinal ganglion cells) in WT and R6/2 retina. DAPI nuclear counterstain is shown in blue. ONL, outer nuclear layer; INL, inner nuclear layer; GCL, ganglion cell layer.

By comparison with cones and ipRGCs, rod photoreceptors and levels of rhodopsin staining appeared normal in R6/2 mice at time points far beyond those at which downregulation of cone opsin and melanopsin expression was observed (Fig. 6A). Levels of rhodopsin staining were similar to WT controls up to 15 weeks. Signs of rod degeneration were, however, evident in R6/2 mice at 20 weeks, although this is likely to be secondary to the significant anatomical remodelling of the retina layers that occurs by this time point (only minimal levels of retinal dysplasia were observed prior to 15 weeks of age). Similarly, levels and patterning of calbindin staining (labels horizontal cells and a subset of amacrine cells; Fig. 6A) and PKCα staining (labels ON bipolar cells; Fig. 6B) appeared normal in R6/2 mice at all time points, with a disruption in the patterning of staining only evident at 20 weeks due to extensive retina dysplasia. Whilst there was a slight reduction in levels of tyrosine hydroxylase (TH) mRNA (Supplementary Material, Fig. S5C), there was no difference in levels of TH staining detected within dopaminergic amacrine cells of the R6/2 retina (Fig. 6B, Supplementary Material, Fig. S5F). The levels of Brn3a expression, and the total number of RGCs also appeared normal between R6/2 and WT mice at all time points (Fig. 6C).

### Immunostaining of rods, cones and melanopsin in Q175 mice

**Figure 7.**
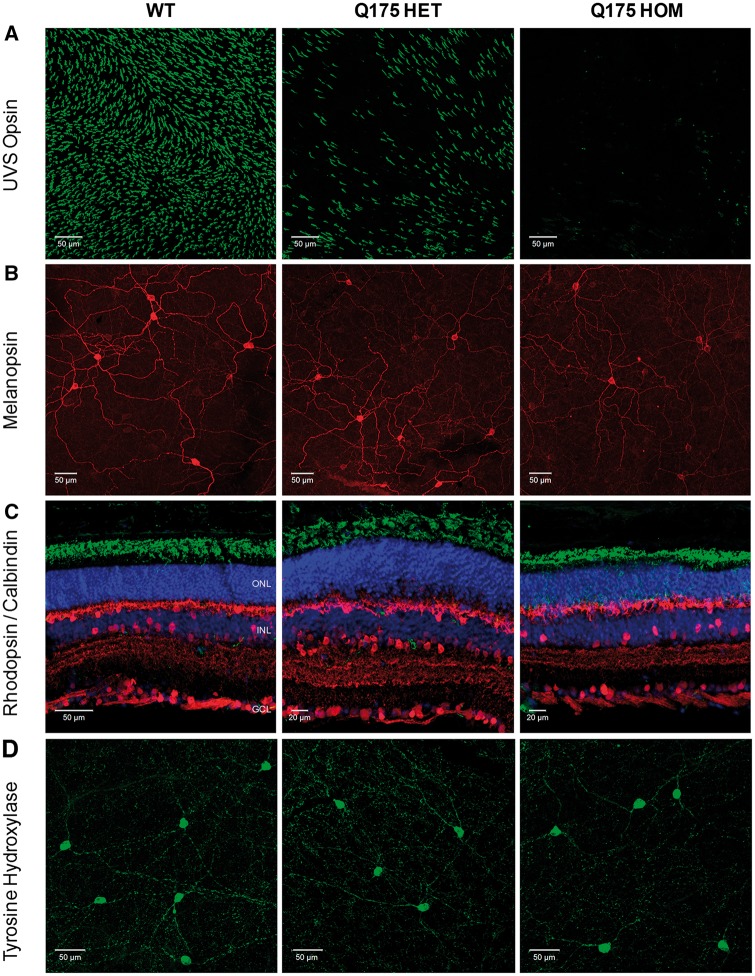
Downregulation of cone opsin and melanopsin expression in Q175 retina at late stage of disease. (**A**) Images showing the levels of UVS cone opsin observed in flatmount retina from WT, Q175 heterozygous (HET) and Q175 homozygous (HOM) mice at 20 months of age. All images collected from the ventral retina where all cones express UVS opsin. (**B**) Images showing the levels of melanopsin observed in flatmount retina from WT, Q175 HET and Q175 HOM mice at 20 months of age. All images collected from the ventral retina. (**C**) Images showing levels of anti-rhodopsin (green, labels rods) and anti-calbindin labelling (red, labels horizontal cells and a subset of amacrine cells). (**D**) Images showing levels of anti-tyrosine hydroxylase labelling (green, marker of dopaminergic amacrine cells) observed in flatmount retina from WT, Q175 HET and Q175 HOM mice at 20 months of age. DAPI nuclear counterstain is shown in blue. ONL, outer nuclear layer; INL, inner nuclear layer; GCL, ganglion cell layer. Images showing the levels of anti-UVS cone opsin, anti-MWS opsin, anti-protein kinase-C alpha (PKCα), and anti-Brn3a labelling are shown in Supplementary Material, Fig. S7. Loss of PNA labelling in Q175 HOM mice at 20 months is shown in Supplementary Material, Fig. S8.

As seen in R6/2 mice, a complete loss of UVS opsin, MWS opsin and cone arrestin expression was evident in the retina of Q175 HOM mice at 20 months (Fig. 7A; Supplementary Material, Fig. S7A and B), with widespread cone degeneration observed in Q175 HOM mice at this time point as evident by a loss of PNA labelling (Supplementary Material, Fig. S8). Q175 HOM mice also showed a downregulation of melanopsin expression, although this effect was less obvious than that observed for R6/2 mice (Fig. 7B, Supplementary Material, Fig. S9). Interestingly, Q175 HET mice showed an intermediate phenotype, with an incomplete loss of UVS opsin and cone arrestin staining evident in these mice at similar ages (Fig. 7A; Supplementary Material, Fig. S7A and B), and only a subtle reduction in levels of melanopsin expression (Fig. 7B; Supplementary Material, Fig. S9). There were also clear examples of dysplasia in retina from 20 month old Q175 HOM mice but not in Q175 HET mice, although this was less severe than that seen in R6/2 mice (for example see Supplementary Material, Fig. S7C). Rhodopsin staining was normal in Q175 HET and Q175 HOM mice (Fig. 7C), as were levels of calbindin (horizontal cells and amacrine cells) (Fig. 7C), PKCα (ON bipolar cells) (Supplementary Material, Fig. S7C) and Brn3a labelling (RGCs) (Supplementary Material, Fig. S7D). There was no difference in levels of TH staining detected within dopaminergic amacrine cells of Q175 HOM retina (Fig. 7D; Supplementary Material, Fig. S9), although similarly to R6/2 mice, a subtle reduction in levels of TH mRNA were detected in Q175 HOM mice (Supplementary Material, Fig. S9).

## Discussion

HD mouse models recapitulate the circadian abnormalities seen in patients. In this study, we found that both R6/2 and Q175 mice had a progressive attenuation of the PLR (that we used as a marker of ipRGC activity), accompanied by a range of morphological abnormalities in the retina. These included a downregulation of melanopsin and cone opsin expression, and ultimately degeneration of cones but not of ipRGCs. Since ipRGCs play a primary role in mediating both the PLR and circadian photoentrainment, the retinal deficits found in R6/2 and Q175 HD mice may contribute to the circadian breakdown seen in HD.

R6/2 mice show a rapid progression of disease, with circadian abnormalities appearing from the age of 10–12 weeks and resulting in a complete disintegration of rest-activity rhythms by ∼15–16 weeks and an early death at ∼24 weeks (43). The PLR deficit was mild at 9 weeks (presymptomatic stage), and worsened by 12 weeks, the age at which the R6/2 mice start to become arrhythmic. PLR deficits were seen in the Q175 HOM mice by the age of 20 months, at which time abnormalities but not complete breakdown of rest-activity rhythms are also seen (44). By contrast, 20 month old Q175 HET mice still had no apparent deficits in their PLR compared to the age-matched WT mice, an age at which they do not show major circadian abnormalities.

Both melanopsin, and rod/cone-based signals contribute to driving the PLR. Transgenic mice without functioning rods and cones and lacking melanopsin, lose the ability to constrict their pupil in response to light (24). By contrast, mice lacking both rods and cones but with a functional melanopsin system still maintain the PLR, albeit with a reduced sensitivity (45). Mice lacking only melanopsin show a PLR similar to WT mice at dim light irradiance, but incomplete PLR at high irradiance, failing to reach full pupil constriction to bright light (19). In R6/2 mice, we observed attenuated PLR at low and moderate irradiances at 12 and 15 weeks (an indication of rod/cone deficits), and a failure to reach full pupil constriction observed in 20 week old R6/2 mice. These data suggest that at 20 weeks of age, the phenotype of R6/2 mice is similar to that seen in melanopsin knockout mice, with a failure to reach full constriction in response to bright light (19).

With respect to circadian defects, the loss of either rods, cones or melanopsin would be expected to result in only subtle circadian phenotypes, as these different photoreceptors perform overlapping roles in providing light input for non-image forming responses (46–48). As ipRGCs provide the principal conduit for rod- and cone-driven inputs to the SCN, downregulation of melanopsin does not preclude rods and cones driving non-image forming responses to light. Our analysis of the HD retina revealed a range of morphological abnormalities in the retina of R6/2 mice, with the earliest pathology observed being the loss of proteins necessary for normal cone function, including cone opsins and cone arrestin (necessary for termination of cone light responses). We observed a reduction of rhodopsin expression and disruption of rod photoreceptors only at the latest age points, with these changes only evident following the onset of retina dysplasia. Previous studies have suggested that the dark adapted PLR is primarily dependent upon an interaction between cones and melanopsin, with cones mediating responses at lower irradiances and melanopsin primarily contributing at higher irradiances (25). Our data are consistent with this model, with PLR defects at low irradiances explained by the marked degeneration of cone opsin, whereas melanopsin defects become apparent at higher irradiances in older mice. Previous studies have shown that classical photoreceptors play a role in regulating melanopsin expression (49–51). As such, the loss of cone function could plausibly account for the changes in melanopsin expression observed. However, aging studies on mice lacking all rods and cones (*rd/rd cl*) show no changes in melanopsin mRNA or levels of immunostaining using the same methods used in this study (52). As such, the dramatic changes we describe here appear unlikely to be due to the loss of cone function alone.

In agreement with a previous study (34), we did not find any changes in the number and morphology of retinal ganglion cells in symptomatic R6/2 mice. We did, however, observe a progressive downregulation of melanopsin expression within ipRGCs of R6/2 retina, including all ipRGC subtypes that are detectable by immunostaining (41).

At 20 weeks of age (end stage), R6/2 mice showed a severe downregulation of melanopsin. This is consistent with the diminished PLR at high irradiance as described above (19). Despite the downregulation of melanopsin expression, there were no observable changes in the morphology of ipRGCs, or evidence of neurodegenerative loss of ipRGCs at any stage of disease studied in R6/2 mice. Moreover, despite the widespread accumulation of Htt aggregates in the retinal ganglion cell layer by 15 weeks of age, ipRGCs contained few, if any, detectable aggregates at this time point. However, aggregates were commonly detected within ipRGCs at 20 weeks, corresponding to the age at which impaired maximum pupil constriction is evident in R6/2 mice. The preservation of ipRGCs could be explained by a specific resistance to neurodegeneration caused by alterations in mitochondrial function (53,54), which play major roles in the pathogenic processes in HD (55).

The loss of cones and reduction in melanopsin expression observed in R6/2 mice was recapitulated in Q175 mice. The combined downregulation of cone opsin and also melanopsin expression represents a ‘double hit’ loss-of-function, and may combine to contribute to phenotypes observed in R6/2 and Q175 mice. However, the progression and severity of PLR phenotypes observed in R6/2 and Q175 mice may potentially be explained better by the timeline of melanopsin downregulation in these mouse models than by the loss of cone opsin. Loss of cone opsin is complete in both mouse models, yet Q175 mice have a more subtle phenotype consistent with the more subtle loss in levels of melanopsin expression.

Together, our results suggest that retinal degeneration, and in particular the marked deterioration of cone and melanopsin pathways in HD mice likely result in reduced retinal photoreception and changes in levels and properties of light information transmitted from the retina. These changes may contribute to the progressive dysregulation of circadian rhythmicity seen in HD. As the disease progresses, the circadian system of R6/2 and aged Q175 HOM mice becomes increasingly insensitive to the external light-dark (LD) cycle, particularly when the environmental light levels are low. Consistent with these defects in light input, bright light therapy was able to reverse the rest-activity rhythm dysfunction in R6/2 mice (31).

Dopamine is present in the retina, produced by a subclass of amacrine cells where it is involved in neural processes for light adaptation (56). Since there is evidence to suggest that melanopsin expression and levels of dopamine are interdependent in the retina (57–59), it was tempting to suggest that an imbalance of the retinal dopaminergic system causes the downregulation of melanopsin expression. For both R6/2 and Q175 mice, however, we found that dopaminergic amacrine cells and levels of TH expression were unaffected by disease progression, although subtle reductions in levels of TH mRNA expression were observed for both R6/2 and Q175 mice. Further investigations are needed to understand why melanopsin expression is specifically and progressively reduced in R6/2 and Q175 mice.

Some studies have reported abnormal cone function and colour vision in HD patients (39,40), suggesting the involvement of the retina in the process of HD degeneration. To our knowledge, however, none has reported a link between retinal dysfunction and circadian abnormalities. In other neurodegenerative disease such as Parkinson’s disease (PD) and Alzheimer’s disease (AD), there is evidence for a contribution of retinal deficits to circadian abnormalities (60–62). For example in AD, a *post mortem* study of retina revealed a specific pathology and loss of ipRGCs associated with β-amyloid deposits (61), suggesting that ipRGC degeneration contributes to circadian dysfunction (62). Therefore, the retina may be a tissue of interest in HD to assess not only the role of melanopsin expression and circadian photoreception in the pathogenesis of circadian abnormalities but also the possibility of using circadian photoreception as a marker of disease progression.

Finally, a bright light therapy has beneficial effects for patients with AD (63) and PD (64) but to date this has not been investigated in HD patients. Given that bright light therapy effectively delays the deterioration of circadian rhythms in HD mice (31), this might be a promising non-pharmaceutical strategy to improve rest-activity rhythms and thus the quality of life of HD patients.

## Materials and Methods

### Animals

Experiments were conducted under the UK Animals (Scientific Procedures) Act 1986 with the approval of the University of Cambridge License Review Panel. R6/2 Q250 (10 males and 10 females) and WT (10 males and 10 females) mice were taken from a colony established in the University of Cambridge (CBA x C57Bl/6J background). WT (18 males and 2 females) and Q175 mice (28 males and 28 females) on a C57Bl/6J background were obtained from a colony established in the University of Cambridge, which originated from founders obtained from the Jackson Laboratory (Bar Harbor, Maine, USA). Genotyping and determination of the CAG repeat length were carried out on tail biopsies by Laragen (Los Angeles, USA) using GeneMapper. R6/2 Q250 males had a mean of CAG repeat length of 249 ± 1 and females 248 ± 1. Q175 HET for the mutation (*n =* 35) had a mean CAG repeat length of 166 ± 1 and HOM for the mutation (*n =* 25) had a mean CAG repeat length of 159 ± 1 for the shorter allele and 173 ± 1 for the long allele.

### Husbandry

Prior to the experiments, mice were kept in age-matched groups of up to 10 animals of the same sex and genotype for R6/2 mice and of the same sex and mixed genotype for Q175 mice. Mice were kept in a controlled environment with 12:12 LD cycle, at a room temperature of 21–23 °C and humidity of 55% ± 10. Mice had free access to dry laboratory food and water in lowered bottles with elongated spouts. The mice were transferred to clean cages twice weekly.

### Pupillometry

The pupil light reflex is the physiological response that regulates the amount of light reaching the retina by changing the pupil diameter. Prior to the pupil light reflex test, mice were dark adapted in constant darkness in a ventilated circadian cabinet (Scanbur, Denmark) for at least 1 h. The pupillometry testing was conducted in the light period of a 12:12 LD cycle between ZT4 and ZT8 (4–8 h after the lights onset).

R6/2 and Q175 mice were tested at different stages of the disease. A group of R6/2 mice (10 males and 10 females for each group) was tested longitudinally at 5 different ages that represented different stages of disease; a presymptomatic stage (9 weeks), early symptomatic stage (12 and 15 weeks) and late stage of the disease (18 and 20 weeks) to study progressive phenotype.

Q175 mice were tested cross-sectionally. Q175 mice (*n =* 76) were distributed into 3 experimental groups by age and tested at 9 months (presymptomatic; 6 WT, 8 HET and 6 HOM mice), 15 months (early symptomatic; 7 WT, 12 HET and 9 HOM mice) and 20 months (symptomatic; 7 WT, 12 HET and 9 HOM mice).

Monochromatic light (470 nm) delivered by a LED light source (Luxeon Rebel LED assembly, SR-01-B0040) was used to stimulate one eye, and pupillary light responses under bright light (14.6 log quanta/cm^2^/s) were measured from the other eye. Lower irradiances (12.9, 11.6, 10.5 and 9.3 log quanta/cm^2^/s) were tested by positioning corresponding neutral density filters into the light path. The irradiances were measured and verified with a radially calibrated spectrophotometer (Ocean Optics, UK). Using routine laboratory handling techniques as previously described (65), the mice were ‘scruffed’ and placed onto a platform. The mice were positioned so that one eye received the LED light and the pupil response in the contralateral eye was recorded by an IR-sensitive CCD camera (Prosilica, GC1020). The camera was connected to a PC computer with custom-made pupillometry software (Pupil6.3.vi running in LabView) which set up the recording of the consensual pupil response to the light stimulus by 5 frames per second, and controlled the duration of the light delivery as follows: 2 s in dark (pre-stimulus), 10 s of light (stimulus) followed by 17 s of dark (post-stimulus, recovery). The pupil images were collected by the software controlling stimulus presentation. The images were analysed with ImageJ (http://rsbweb.nih.gov/ij/), allowing pupil area measurement for each image. In some cases, the pupil was not entirely visible (due to whiskers cutting the pupil area or ptosis in symptomatic HD mice); this prevented the use of the automatic analysis. For these cases, pupil diameter was manually analysed using ImageJ. The responsive pupil areas or diameter were calculated as a percentage of the baseline pupil area or diameter (when fully dilated under dark).

Only results from animals for which we had full data for the five irradiances tested are presented. For R6/2 mice, data shown at 9 weeks are from 19 R6/2 and 20 WT mice; at 12 weeks from 20 R6/2 and 20 WT mice; at 15 weeks from 16 R6/2 and 20 WT mice; at 18 weeks from 20 R6/2 and 19 WT mice, and at 20 weeks, from 19 R6/2 and 19 WT mice. For Q175 mice, data shown at 9 months are from 6 WT, 8 HET and 6 HOM mice; at 15 months from 6 WT, 12 HET and 9 HOM mice; and at 20 months from 7 WT, 12 HET and 8 HOM mice. Data from male and female R6/2 mice were originally analysed separately. We found that while the magnitude of PLR deficit was slightly greater in females than in R6/2 males (compared to control sex-matched mice), the PLR deficits had the same trends. We therefore pooled the data from both sexes for clarity of presentation.

### Drugs

At 5 to 10 min prior to PLR testing, mice received a topical application of tropicamide 1% (Sigma) to prevent constriction in the stimulated eye (45). Consensual pupil constriction was measured in the contralateral (undilated) eye. Carbachol (carbamylcholine chloride, Sigma) was prepared at the concentration of 100 mM in 1X phosphate-buffered saline (PBS). Carbachol induces pupil constriction in normal mice and was tested in R6/2 and Q175 mice as a control to verify whether or not the animals have an intrinsic defect in the iris sphincter pupillae muscles that are responsible for pupil constriction (45). The drug was applied topically to the cornea of the mouse eye with a microtip transfer pipette and the pupil response was assessed in darkness, before, and 10 min after drug application.

### Immunocytochemistry

Four groups of R6/2 and age-matched WT mice were killed at 9, 12, 15 and 20 weeks (*n =* 7–8); Q175 mice and WT littermates (*n =* 6 for each genotype) were killed at ∼20 months. All the mice were killed at the same time of day, between ZT7 and ZT8, in order to match the time window at which PLR tests were performed (ZT4-ZT8). Preparation and immunostaining of retina sections and whole retina flatmounts was performed as described previously (41,66). We used primary antibodies against melanopsin (as a marker of ipRGCs), UVS and MWS cone opsins (as markers of cones), rhodopsin (as a marker of rods), TH (as a marker of dopaminergic amacrine cells), PKCα (as a marker of ON bipolar cells), calbindin (as a marker of horizontal cells and a subset of amacrine cells), Brn3a (as a marker of retinal ganglion cells immunonegative for melanopsin), cone arrestin (a marker of cones), and MW8 (as a marker of Htt aggregates). Primary antibodies were incubated for 24 h (sections) or 72 h (retina flatmounts) at 4^°^C, PNA lectin (specifically labels cone cell membranes) conjugated to Alexa-568 (Thermo Fisher) was incubated at 50 µg/ml for 2 h at room temperature. For staining with the MW8 antibody, in an attempt to reduce ‘mouse on mouse’ background staining, sections were first incubated with unconjugated donkey anti-mouse monovalent Fab fragments (Jackson Immunoresearch) 1:100 for 2 h at RT to block endogenous IgGs present in the mouse tissue. Secondary antibodies were incubated at 1:200 for 2 h at room temperature (sections) or 24 h at 4^°^C (flatmounts). All secondary antibodies were raised in donkey and conjugated with Alexa dyes (Thermo Fisher). A summary of the different antibodies and dilutions used are shown in Table 1. For retinal sections, all antibodies were diluted in PBS with 2.5% donkey serum and 0.2% Triton-X. All wash steps were performed using PBS with 0.05% Tween-20. For staining of retina flatmounts, levels of Triton-X were increased to 1%. Samples were mounted in Prolong Gold anti-fade media containing DAPI (Thermo Fisher).

**Table 1.** **** Antibodies used in the immunohistochemical study

Target	Antibody species	Antibody/Source	Dilution	Secondary antibody
Melanopsin	Rabbit polyclonal	UF006, Advanced Targeting Systems	1:2500	Donkey anti-rabbit Alexa 488
UVS opsin	Goat polyclonal	sc-14363, Santa Cruz Biotech	1:1000	Donkey anti-goat Alexa 488
MWS opsin	Rabbit polyclonal	JH492, Gift J. Nathans	1:1000	Donkey anti-rabbit Alexa 568
Rhodopsin	Mouse monoclonal	ID4, Gift Ji. Cowing	1:5000	Donkey anti-mouse Alexa 488
TH	Chicken polyclonal	ab76442, Abcam	1:1000	Donkey anti-chicken Alexa 488
PKC alpha	Rabbit polyclonal	ab32376, Abcam	1:1000	Donkey anti-rabbit Alexa 568
Calbindin	Rabbit polyclonal	ab11426, Abcam	1:1000	Donkey anti-rabbit Alexa 568
Brn3a	Goat polyclonal	sc-31985, Santa Cruz Biotech	1:1000	Donkey anti-goat Alexa 488
Cone arrestin	Rabbit polyclonal	ab15282**,** Millipore	1:1000	Donkey anti-rabbit Alexa 568
MW8	Mouse monoclonal	Developmental Studies Hybridoma Bank	1:1000	Donkey anti-mouse Alexa 488

### Quantification of immunofluorescence

Quantification of immunofluorescence levels in confocal images collected from whole retina flatmounts was performed using ImageJ software (NIH; rsbweb.nih.gov/ij/). In order to ensure all images were processed under identical conditions all individual images for comparison were first compiled into an image stack. Background subtraction and then threshold analysis were performed on all images within the stack using automated settings within ImageJ. For analysis of cone opsin staining the percentage area of each image deemed above threshold was calculated to provide a measure of the retina area covered by UVS opsin. Due to the lower density of ipRGCs and TH positive amacrine cells, the mean greyscale values of each image area above threshold were calculated and used as a quantitative measure of intensity of immunofluorescence signal. All measurements were performed using default settings in ImageJ. Images were collected from *n =* 3–4 retina per group, with multiple images collected from each retina averaged to produce a single value per retina. Images used for quantification were typically acquired at low magnification (x20 objective) to maximise the area of the retina used for analysis. All images were collected from the ventral retina. Data are shown normalised to values calculated for WT retina at 9 weeks.

### qPCR

Following enucleation, whole eyes were stored at −80^°^C prior to use. Tissue was homogenised in TRIzol Reagent (Thermo Fisher) and total RNA isolated using RNeasy spin columns (Qiagen) with on column DNase treatment (Qiagen). 1µg of total RNA was reverse transcribed using SuperScript III with oligo(dT)_20_ primers (Thermo Fisher) and qPCR performed using Quantifast SYBR Green PCR mastermix (Qiagen) on a StepOne thermal cycler (Applied Biosystems). Quantification of transcript levels was performed using a comparative threshold cycle approach (67) with levels of target gene expression normalised to the geometric mean expression of three house-keeping genes (Arp, Tbp and Gapdh). Primer sequences were as follows; UVS F; TCTTCACAGTCTTCATCGCCAGC; UVS R; TTCAAAAGCCAGGAAAGCCAATG; Opn4 F; TCACAGGGATGCTGGGCAATC; Opn4 R; TTCTTGTAGAGGCTGCTGGCAAAG; Thy-1F; TGCTGTTCTCAACCTGCTCTTCTCC; Thy-1R, GGGTCTCTAAGTGGTGGATTTTGGC; Arp F; CGACCTGGAAGTCCAACTAC; Arp R; ATCTGCTGCATCTGCTTG; Tbp F; TGGGCTTCCCAGCTAAGTTC; Tbp R; GGAAATAATTCTGGCTCATAGCTACTG; Gapdh F; TGCACCACCAACTGCTTAG; Gapdh R; GATGCAGGGATGATGTTC.

### Image acquisition

Fluorescent images were collected using an LSM 710 laser scanning confocal microscope and Zen 2009 image acquisition software (Zeiss). Individual channels were collected sequentially. Laser lines for excitation were 405 nm, 488 nm, and 561 nm. Emissions were collected between 440 and 480, 505 and 550, and 580 and 625 nm for blue, green and red fluorescence respectively. For all images, global enhancement of brightness and contrast was performed using Zen Lite 2011 image analysis software (Zeiss). For direct quantitative comparisons (where stated), all images were acquired and processed under identical conditions.

### Statistics

Statistical analyses were performed using Statistica 19.0 software (version 12, StatSoft Inc., Tulsa, USA) or Prism 5 (GraphPad Software Inc., San Diego, USA). Analysis of variance (ANOVA) with repeated measures was performed to investigate differences between groups, followed by a *post hoc* Student Newman Keuls test when a main effect was found. The results were considered significant when *P <* 0.05.

## Supplementary Material

Supplementary Material is available at *HMG* online.

## Supplementary Material

Supplementary Data
