# Supplementary material for: Attenuated pupillary light responses and downregulation of opsin expression parallel decline in circadian disruption in two different mouse models of Huntington’s disease
Source: Hum Mol Genet. 2016 Nov 27;25(24):5418–32. doi: 10.1093/hmg/ddw359 (PMC5418835; doi:10.1093/hmg/ddw359)
Supplement: Supplementary Data [file ddw359_Supp.pdf]

Attenuated pupillary light responses and downregulation of opsin expression parallel decline in circadian disruption in two different mouse models of Huntington's disease

Koliane Ouk[1§], Steven Hughes[2§], Carina A. Pothecary[2], Stuart N. Peirson[2] and A. Jennifer Morton[1*]
[§] These authors contributed equally to this work.

Supplementary material

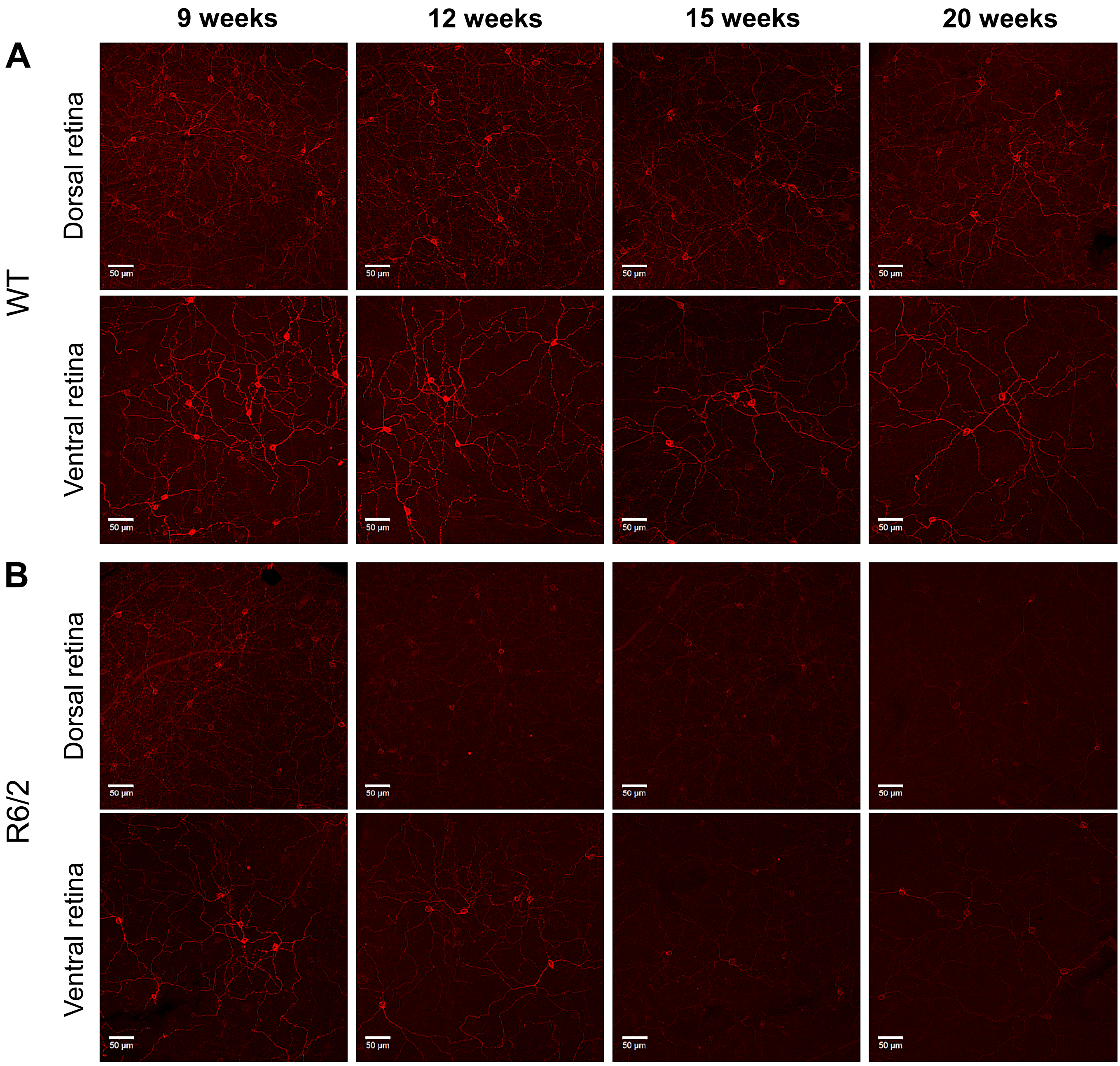

Supplementary Figure 1. Levels of melanopsin expression in R6/2 retina at 9, 12, 15 and 20 weeks of age. Images showing the levels of melanopsin labelling (red) observed in dorsal and ventral regions of flatmount retina from wild type (WT) (A) and R6/2 mice (B) at 9, 12, 15 and 20 weeks of age.

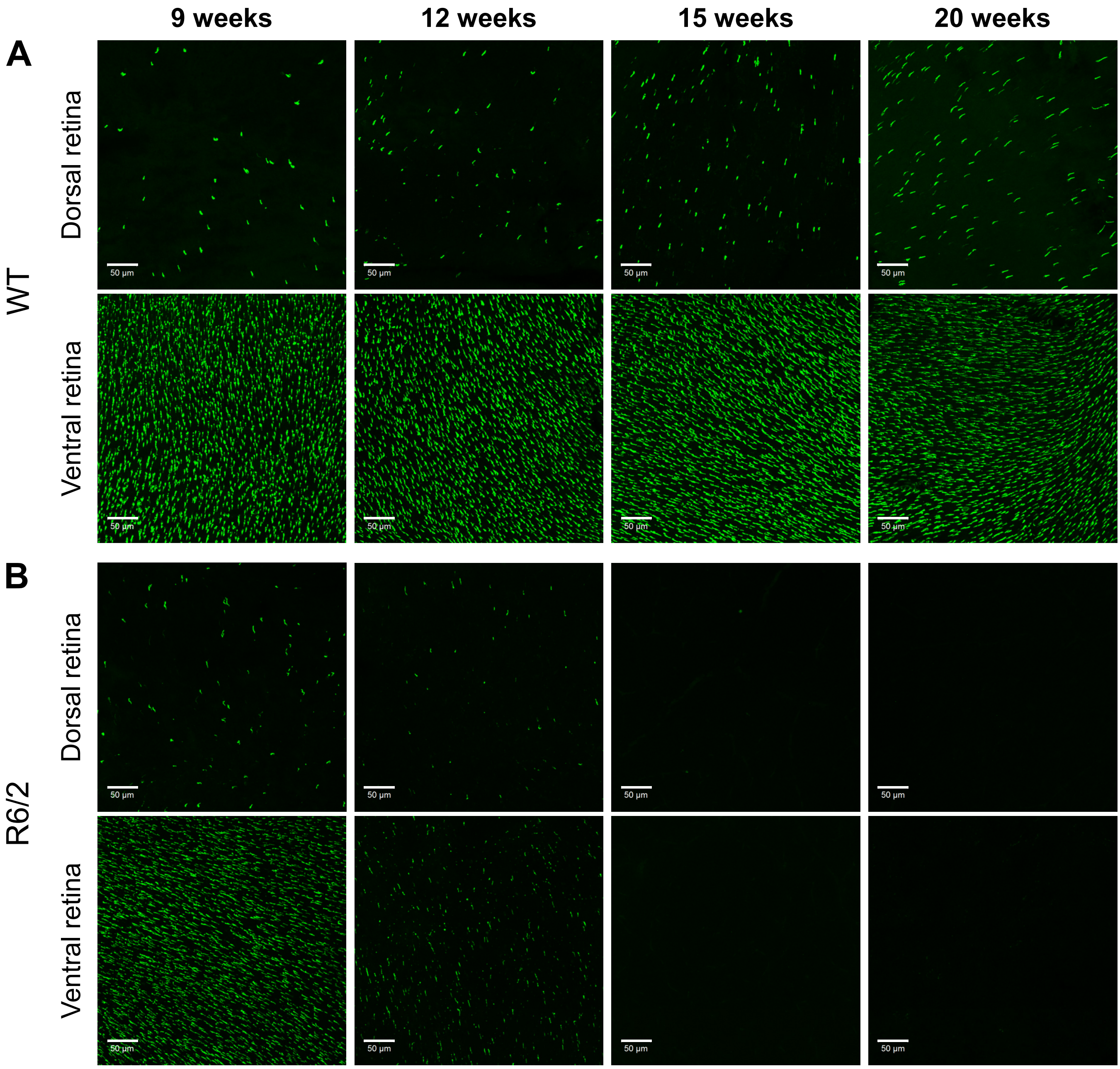

Supplementary Figure 2. Levels of cone opsin labelling observed in R6/2 retina at 9, 12, 15 and 20 weeks of age. Images showing the levels of UVS cone opsin (green) observed in dorsal and ventral regions of flatmount retina from wild type (WT) (A) and R6/2 mice (B) at 9, 12, 15 and 20 weeks of age. In the ventral retina both M-cones and S-cones express UVS opsin, whereas only S-cones express UVS opsin in the dorsal retina.

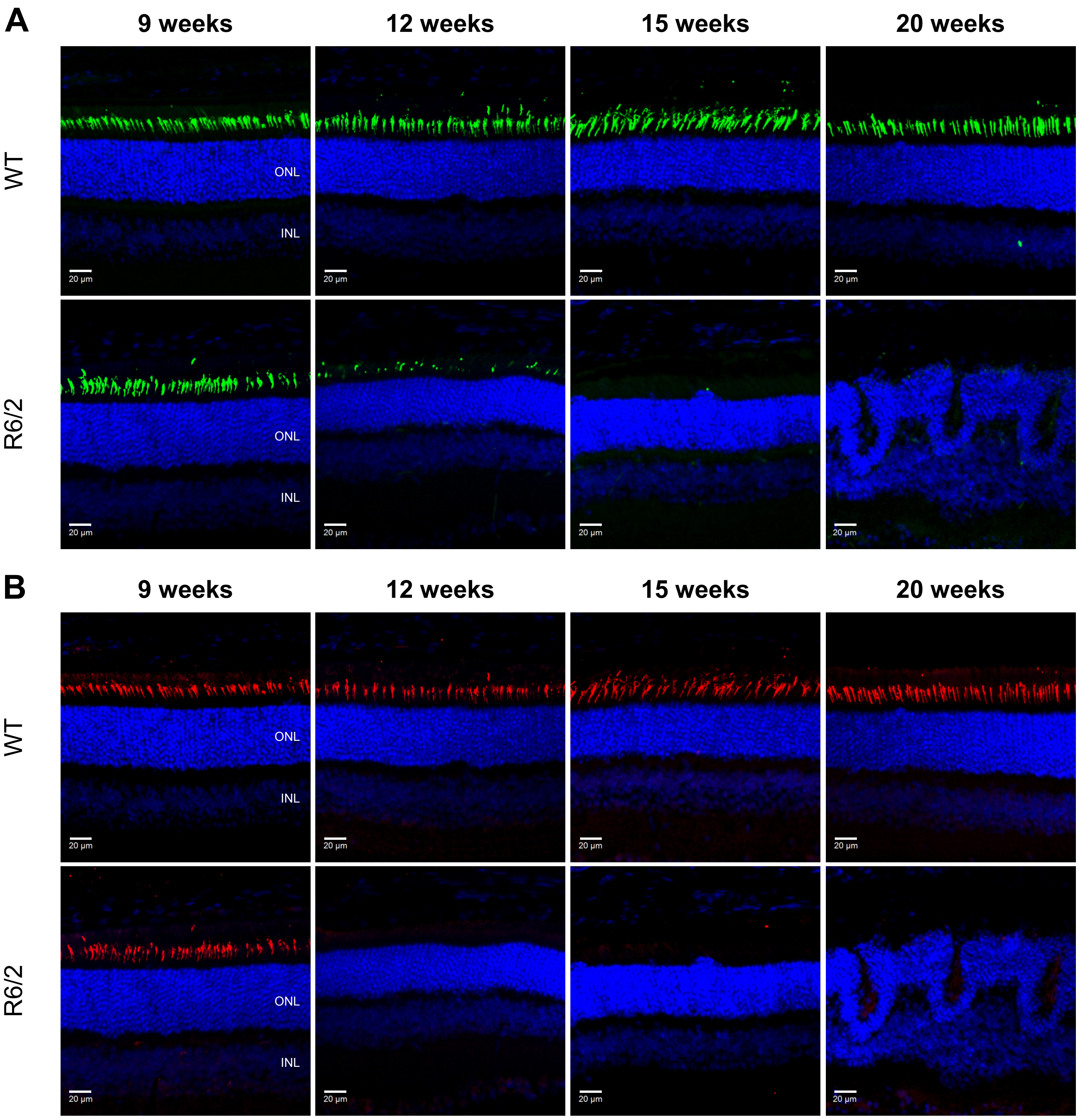

Supplementary Figure 3. Levels of UVS cone opsin and MWS cone opsin expression in R6/2 retina at 9, 12, 15 and 20 weeks of age. (A) Images showing levels of UVS cone opsin (green) observed in wild type (WT) and R6/2 mice at 9, 12, 15 and 20 weeks of age. (B) Images showing levels of MWS cone opsin (red) observed in WT and R6/2 mice at 9, 12, 15 and 20 weeks of age. All images are collected from the ventral retina where M-cones express both UVS and MWS cone opsin and S-cones express only UVS cone opsin. DAPI nuclear counter stain is shown in blue. ONL, outer nuclear layer; INL, inner nuclear layer.

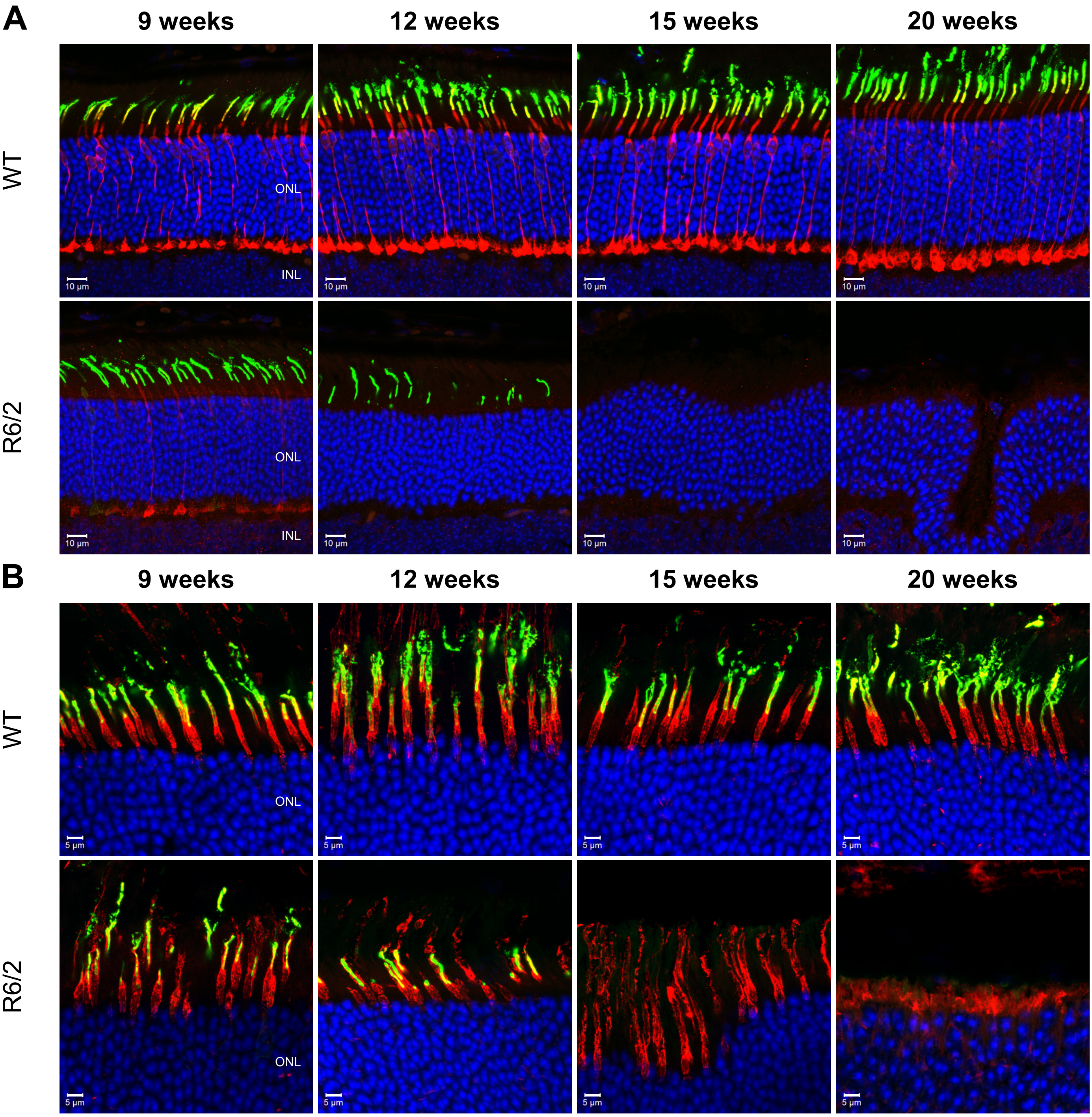

Supplementary Figure 4. Loss of cone arrestin and cone opsin expression in R6/2 retina is observed prior to degeneration of cone photoreceptors. (A) Images showing levels of UVS cone opsin (green) and cone arrestin (red) detected in wild type (WT) and R6/2 mice at 9, 12, 15 and 20 weeks of age. Note that levels of cone arrestin are reduced in R6/2 retina at 9 weeks compared to WT retina, and that loss of detectable cone arrestin expression precedes the loss of UVS cone opsin labelling in R6/2 retina. (B) Images showing levels of UVS cone opsin (green) and peanut agglutinin (PNA) lectin labelling (red) observed in WT and R6/2 mice at 9, 12, 15 and 20 weeks of age. Note that labelling of cone cell membranes with PNA lectin is evident in R6/2 mice at 15 weeks after the complete loss of detectable cone opsin expression. Widespread degeneration of cone cell bodies in R6/2 mice is not observed until 20 weeks. All images are collected from the ventral retina. DAPI nuclear counter stain is shown in blue. ONL, outer nuclear layer; INL, inner nuclear layer.

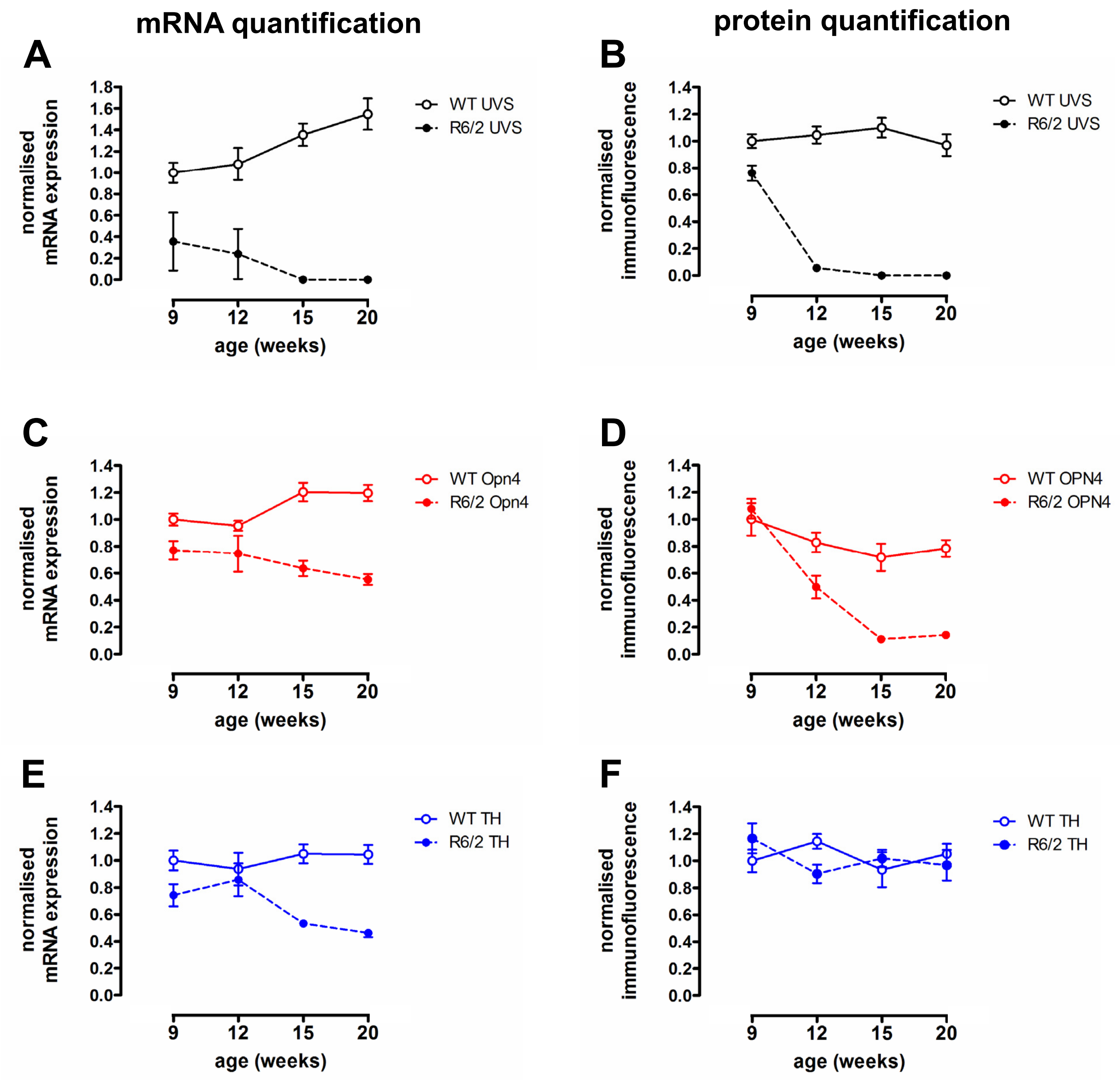

Supplementary Figure 5. Quantification of mRNA and protein levels in R6/2 retina at 9, 12, 15 and 20 weeks of age. (A, C, E) qPCR analysis showing normalised levels of UVS mRNA (A) melanopsin (Opn4) mRNA (C) and tyrosine hydroxylase (TH) mRNA (E) detected in whole eye RNA samples from wild type (WT) and R6/2 mice (n=4-5 samples per group). (B, D, F) Levels of UVS cone opsin protein (B), melanopsin (OPN4) protein (D), and tyrosine hydroxylase (TH) protein detected in WT and R6/2 retina as determined by quantitative analysis of immunofluorescence levels detected in confocal images from retina flatmounts (n=3-4 retina per group). All data are shown normalised to mean values detected for 9 week WT samples. All data is shown as mean ± S.E.M. Statistical analysis was performed using two-way ANOVA. For UVS mRNA, there is a significant effect of genotype ($P<0.0001$), no effect of age ($P>0.05$), and no age x genotype interaction ($P=0.0543$). For Opn4 mRNA, there is a significant effect of genotype ($P<0.0001$), no effect of age ($P>0.05$), and a significant age x genotype interaction ($P=0.0148$). For TH mRNA, there is a significant effect of genotype ($P<0.0001$), no effect of age ($P>0.05$), but a significant age x genotype interaction ($P=0.0213$). For UVS protein, there is a significant effect of age ($P<0.0001$), significant effect of genotype ($P<0.0001$), and significant age x genotype interaction ($P<0.0001$). For OPN4 protein, there is a significant effect of genotype ($P<0.0001$), significant effect of age ($P<0.0001$), and a significant age x genotype interaction ($P=0.0003$). For TH protein, there is no significant effect of genotype ($P>0.05$), no effect of age ($P>0.05$), and no age x genotype interaction ($P=0.1429$).

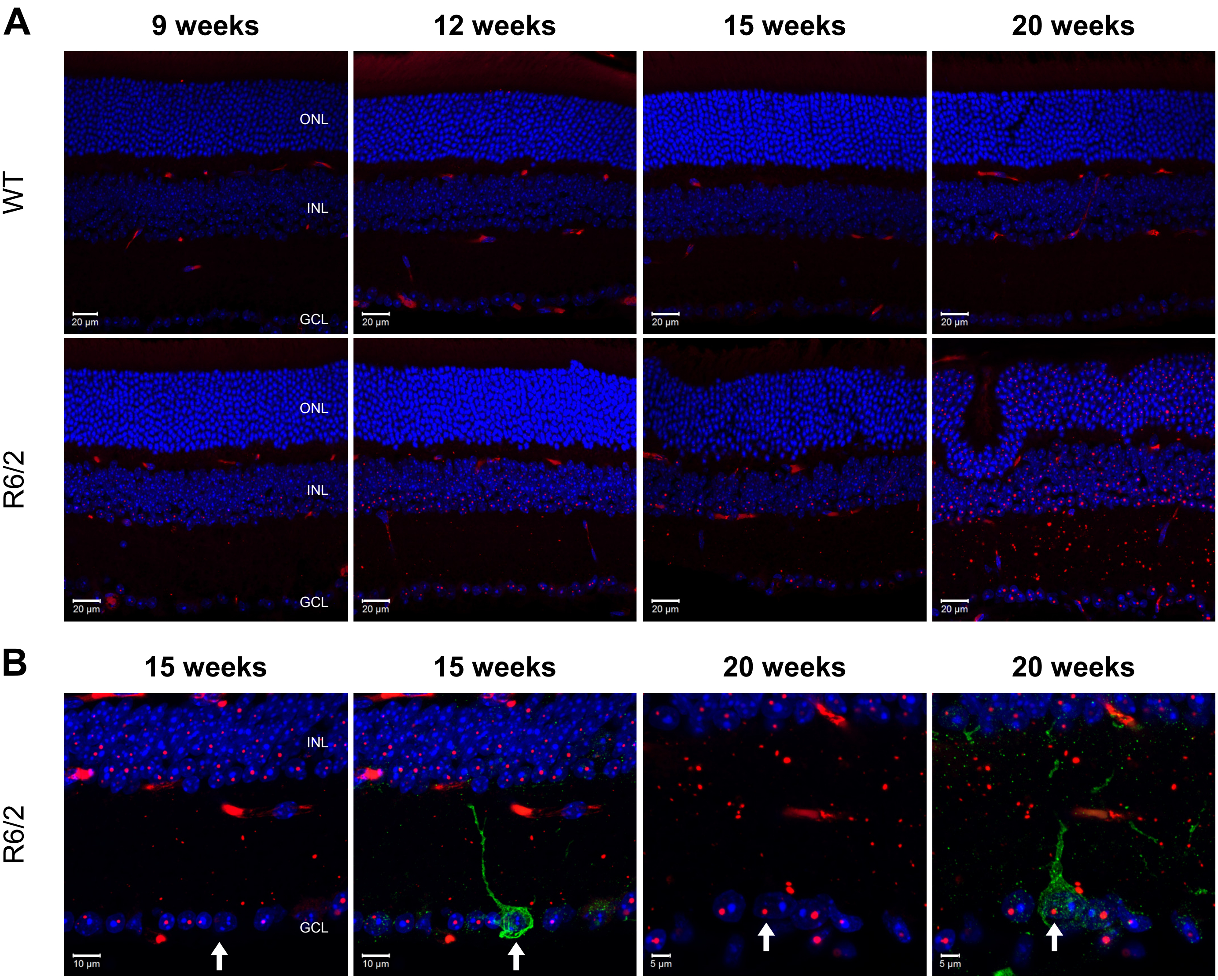

Supplementary Figure 6. Expression of aggregated huntingtin (Htt) protein in the R6/2 retina and ipRGCs at 9, 12, 15 and 20 weeks of age. (A) Images showing levels of aggregated Htt protein (red) observed in wild type (WT) and R6/2 retina at 9, 12, 15 and 20 weeks of age. Note that levels of aggregated Htt detected in R6/2 retina show a steady increase over time, with high levels of aggregated Htt detected in all retinal layers at 20 weeks. Background labelling of blood vessels is observed in both WT and R6/2 retina following staining with the mouse anti-Htt antibody used due to binding of the donkey anti-mouse secondary antibody to endogenous mouse IgGs present in the tissue. (B) Images showing levels of aggregated Htt protein (red) detected within melanopsin expressing ipRGCs (green) of R6/2 retina. Note that Htt labelling is not observed within ipRGS at 9 week and 12 week time points and is almost completely absent from ipRGCs in R6/2 mice at 15 weeks (white arrows) despite the presence of Htt aggregates within the vast majority of cells in the GCL (and other layers) at this time point. Aggregated Htt is however routinely detected within ipRGCs of R6/2 mice at 20 weeks (white arrows). DAPI nuclear counter stain is shown in blue. ONL, outer nuclear layer; INL, inner nuclear layer; GCL, ganglion cell layer.

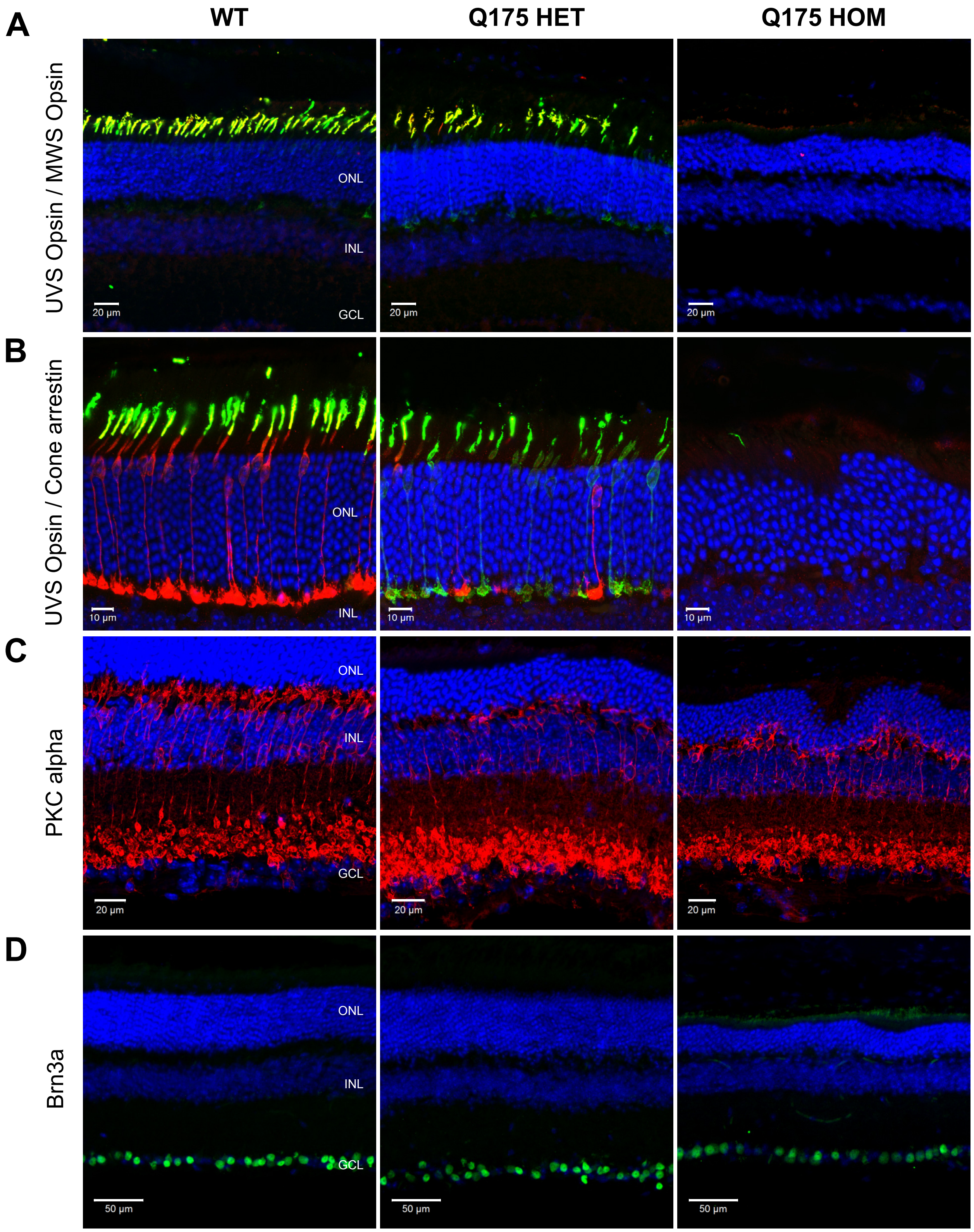

Supplementary Figure 7. Cones, ON bipolar cells, and retinal ganglion cells in the retina of Q175 mice. (A) Images showing the levels of MWS cone opsin (red) and UVS cone opsin (green), (B) UVS opsin (green) and cone arrestin (red), (C) protein kinase-C alpha (PKCα) and (D) Brn3a labelling in wild type (WT), Q175 heterozygous (HET) and Q175 homozygous (HOM) retina at 20 months of age. Note the complete loss of UVS opsin, MWS opsin and cone arrestin labelling in retina of Q175 HOM mice, whereas levels of PKCα and Brn3a are unaffected. Q175 HET mice show an intermediate phenotype with significant but incomplete loss of UVS opsin, MWS opsin and cone arrestin labelling. ONL, outer nuclear layer; INL, inner nuclear layer; GCL, ganglion cell layer.

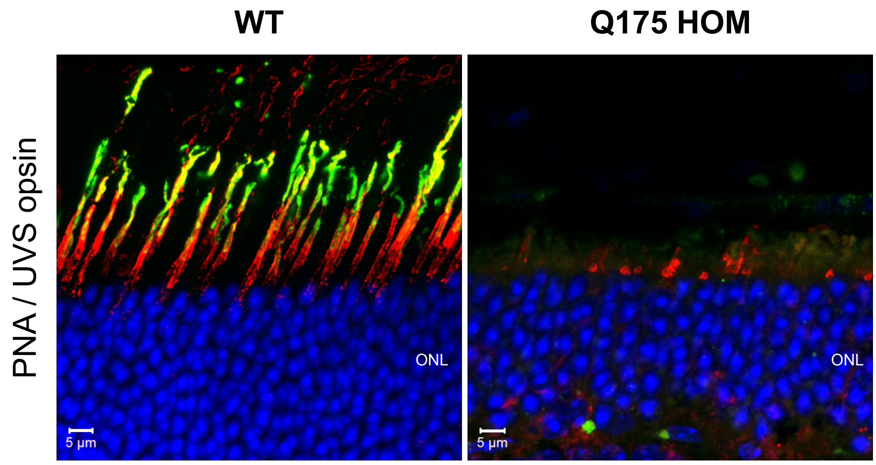

Supplementary Figure 8. UVS cone opsin and PNA labelling of Q175 retina. Images showing levels of UVS cone opsin (green) and peanut agglutinin (PNA) labelling (red) observed in the retina of wild type (WT) and Q175 Homozygous (HOM) mice at 20 months of age. Note the complete loss of detectable cone opsin and PNA labelling in Q175 HOM retina at this age, indicating a complete degeneration of cone photoreceptors. All images are collected from the ventral retina. DAPI nuclear counter stain is shown in blue. ONL, outer nuclear layer.

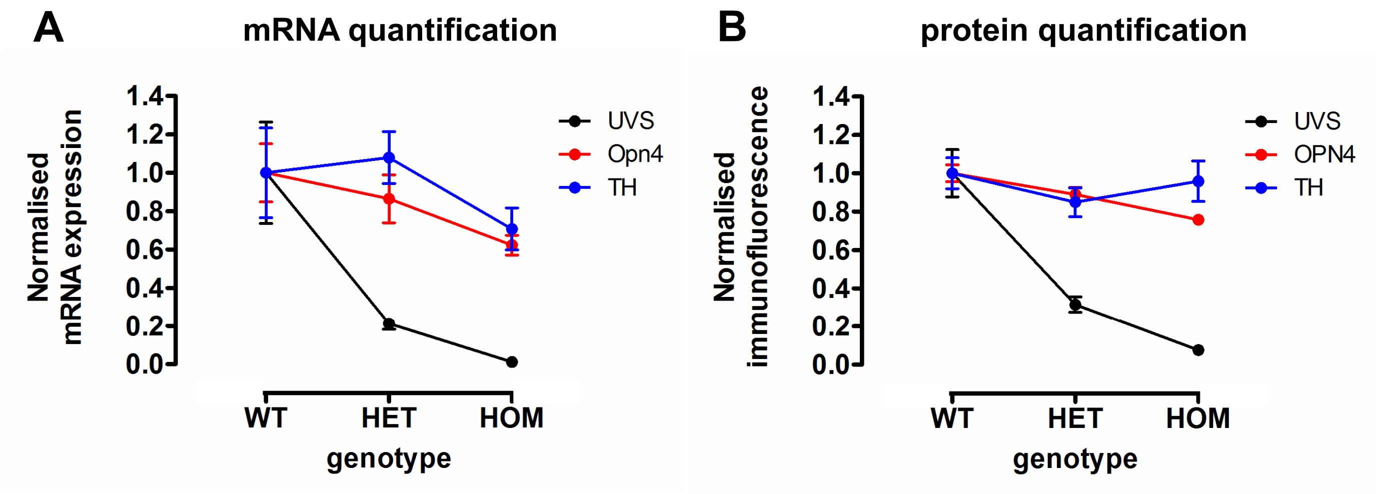

Supplementary Figure 9. Quantification of mRNA and protein levels in Q175 retina. (A) qPCR analysis showing normalised levels of UVS mRNA, melanopsin (Opn4) mRNA, and tyrosine hydroxylase (TH) mRNA detected in whole eye RNA samples from wild type (WT), Q175 heterozygous (HET) and Q175 homozygous (HOM) mice at 20 months of age (n=4-5 samples per group). (B) Levels of UVS cone opsin protein, melanopsin (OPN4) protein, and tyrosine hydroxylase (TH) protein detected in WT, Q175 HET and Q175 HOM retina as determined by quantitative analysis of immunofluorescence levels detected in confocal images from retina flatmounts (n=3 retina per group). All data are shown normalised to mean values detected for WT samples. All data is shown as mean ± S.E.M. Statistical analysis was performed using one-way ANOVA. For TH mRNA, and Opn4 mRNA there is no significant effect of genotype ($P>0.05$). For UVS mRNA, there is a significant effect of genotype ($P=0.0016$), post hoc t-test WT vs HET ($p=0.0179$), WT vs HOM ($P=0.0057$). For TH protein, there is no significant effect of genotype ($P>0.05$). For OPN4 protein, there is a significant effect of genotype ($P=0.0099$), post hoc t-test WT vs HET ($P>0.05$), WT vs HOM ($P=0.0097$). For UVS protein, there is a significant effect of genotype ($P<0.0001$), post hoc t-test WT vs HET ($P=0.0006$), WT vs HOM ($P<0.0001$).
